# *Protaetia brevitarsis* Hydrolysate Mitigates Muscle Dysfunction and Ectopic Fat Deposition Triggered by a High-Fat Diet in Mice

**DOI:** 10.3390/nu17020213

**Published:** 2025-01-08

**Authors:** Kyungeun Park, Sunyoon Jung, Jung-Heun Ha, Yoonhwa Jeong

**Affiliations:** 1 Department of Food Science and Nutrition, Dankook University, Cheonan 31116, Republic of Korea; 2 Research Center for Industrialization of Natural Neutralization, Dankook University, Yongin 16890, Republic of Korea

**Keywords:** *Protaetia brevitarsis* hydrolysates, AMPK activation, metabolic syndrome, high-fat, ectopic fat

## Abstract

Background/Objectives: Obesity is a key factor in metabolic syndrome (MetS) development. Consumption of a high-fat diet (HFD) accelerates the onset of obesity and associated metabolic complications. *Protaetia brevitarsis* (PB) has been traditionally utilized in Korean medicine for its antioxidant, anti-diabetic, anticancer, and hepatoprotective effects. However, specific effects of PB hydrolysate on skeletal muscles have not been fully elucidated. Therefore, this study sought to assess the influence of PB on HFD-induced MetS, focusing on the lipid metabolism and inflammatory responses mediated by AMP-activated protein kinase activation. Methods: To induce obesity, 6-week-old C57BL/6J mice were maintained on an HFD for 8 weeks, after which PB hydrolysate was orally administered for 16 weeks while the HFD regimen was sustained. A glucose tolerance test was conducted orally to evaluate glucose regulation, and forelimb grip strength was assessed upon completion of the experimental period. Histological assessments, serum biochemical analysis, lipid extraction, Western blot analysis, and quantitative reverse-transcription polymerase chain reaction (qRT-PCR) were performed following euthanasia. Results: PB significantly reduced ectopic lipid deposition in skeletal muscles, enhanced muscle strength, and improved insulin sensitivity by increasing fatty acid oxidation via AMP-activated protein kinase/carnitine palmitoyltransferase 1 activation and inhibiting lipogenesis via stearoyl-CoA desaturase 1 gene downregulation. Furthermore, PB alleviated HFD-induced low-grade chronic inflammation by decreasing systemic monocyte chemoattractant protein 1 levels, thereby reducing ectopic fat deposition. Conclusions: This study highlights the potential of PB as a nutraceutical to mitigate MetS in HFD-fed mice.

## 1. Introduction

Obesity is a complex pathological state characterized by abnormal fat accumulation that poses considerable health risks [1]. Global obesity rates are increasing, with estimates suggesting that more than half of the worldwide population will be classified as overweight or obese by 2035 [2]. Obesity contributes substantially to the extreme global burden of chronic diseases and disabilities, affects a wide range of ages and socioeconomic groups, and has profound social and psychological consequences [3]. Obesity is strongly associated with an elevated risk of metabolic disorders, including hypertension, type 2 diabetes mellitus, hyperlipidemia, and cardiovascular disease [4]. An HFD leads to metabolic syndrome, defined by dyslipidemia, insulin resistance, increased oxidative stress, and chronic low-grade inflammation [5].

Chronic overconsumption of energy combined with a sedentary lifestyle facilitates the buildup of white adipose tissue in the abdominal regions, which plays a critical role in the onset of metabolic complications [6]. Adipocytes primarily function as energy storage units by forming triglycerides that regulate energy balance [7]. When fat cell expansion or lipid storage in subcutaneous areas is constrained, ectopic fat tends to accumulate in the visceral adipose tissue and non-adipose organs, including the skeletal muscles, pancreas, and liver. Ectopic fat deposition results in lipotoxic effects and metabolic complications [8]. Skeletal muscle, a metabolically active organ, serves as a reservoir for amino acids and supports protein synthesis and energy production [9]. The skeletal muscle is responsible for nearly 80% of the postprandial clearance of blood glucose [10]. The accumulation of intramuscular adipose tissue (IMAT) impairs muscle function and insulin sensitivity, thereby significantly contributing to metabolic syndrome [11]. Additionally, IMAT-derived pro-inflammatory cytokines negatively affect muscle force and quality [12].

AMP-activated protein kinase (AMPK) is essential for maintaining cellular energy homeostasis, and is activated in response to elevated in AMP-to-ATP and ADP-to-ATP ratios [13]. This enzyme regulates various metabolic pathways and has therapeutic potential for chronic metabolic diseases [14]. In skeletal muscles, AMPK activation facilitates insulin-independent glucose uptake [15] and regulates lipid metabolism through protein phosphorylation and the modulation of gene transcription [16]. For instance, AMPK enhances fatty acid oxidation by phosphorylating acetyl-CoA carboxylase (ACC), reducing malonyl-CoA levels, and accelerating carnitine palmitoyltransferase 1 (CPT-1)-mediated fatty acid transport into the muscular mitochondria [16,17]. Given its pivotal role, AMPK is considered a therapeutic target for metabolic complications [18]. Recent research has focused on its ability to inhibit IMAT accumulation [15,19,20,21].

Several plant-derived compounds, such as resveratrol, quercetin, ginsenosides, curcumin, and berberine, activate AMPK and are beneficial in the treatment of type 2 diabetes and metabolic syndrome [22]. Edible insects contain abundant bioactive nutrients, including polyphenols and flavonoids, which exhibit antioxidant, anti-inflammatory, and insulin-modulating effects [23,24,25]. *Protaetia brevitarsis* (PB) larvae have been approved as an edible ingredient by the South Korean Ministry of Food and Drug Safety, and are rich in amino acids, unsaturated fatty acids, and bioactive compounds, such as polyphenols and flavonoids [26,27]. Various studies have highlighted the anti-obesity [28], anti-inflammatory [29], and anti-diabetic [30] effects of PB, which are mediated by AMPK activation and regulation of lipid metabolism [28,29,31]. Despite its bioactivity, the specific effects of PB on the skeletal muscles remain unknown. We hypothesized that PB hydrolysate modulates AMPK-mediated lipid metabolism, minimizes ectopic lipid accumulation, and improves muscle function in MetS induced by an HFD.

## 2. Materials and Methods

### 2.1. Enzymatic Hydrolysis of PB Powder

PB hydrolysate was prepared via enzymatic hydrolysis to increase its solubility in water and absorption rate [32,33]. Defatted PB powder (Hanmi Nutrition Inc., Paju, Republic of Korea) was mixed with distilled water at a 1 to 10 (*w*/*v*) ratio and subjected to hydrolysis using 1% (*w*/*v*) Alcalase (Novozymes, Bagsvaerd, Denmark) at 60 °C for 5 h while being agitated in a shaking water bath (MaXturdy 45; Daihan Scientific, Wonju, Republic of Korea). The pH of the mixture was modified to 8.0 by adding 1 N NaOH. To stop the enzymatic hydrolysis, the mixture was incubated at 95 °C for 20 min, then centrifuged at 2000× *g* for 15 min. The supernatant was passed through a 6 μm filter paper (Advantec, Tokyo, Japan) with the assistance of a vacuum pump (Gast Manufacturing, Benton Harbor, MI, USA). The filtrate was lyophilized and kept at −70 °C until needed [34].

### 2.2. Animals

**Figure 1:**
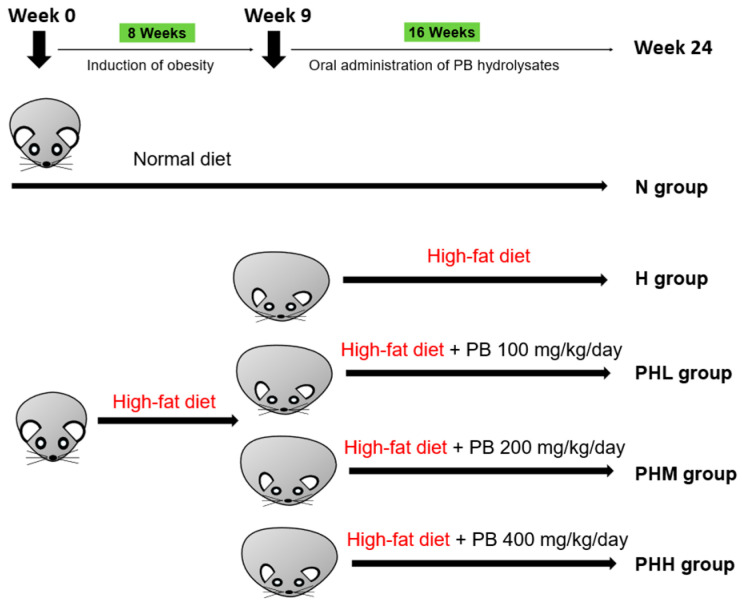
Experimental scheme. PB, *Protaetia brevitarsis*; N, normal chow diet; H, HFD; PHL, HFD + 100 mg/kg PB; PHM, HFD + 200 mg/kg PB; PHH, HFD + 400 mg/kg PB.

Experimental male C57BL/6J mice (5 weeks of age) were purchased from Raon Bio (Yongin, Republic of Korea) and housed (2–3 mice/cage) in a humidity- and temperature-controlled facility (24 ± 2 °C) under a light (12 h)/dark (12 h) cycle, with free access to designated food and water [35]. The mice were ear-tagged, and the cages were labeled with group information for identification. After 1 week of adaptation, the mice (*n* = 66) were randomly assigned to either a normal chow diet (ND) group (*n* = 11; N; 10% kcal from fat; #D12450J; Research Diets, New Brunswick, NJ, USA) or an HFD group (*n* = 55; H; 60% kcal from fat; #D12492; Research Diets). Following eight weeks of obesity induction, overweight mice were randomly separated into four subgroups (*n* = 11/group): HFD (H), HFD + 100 mg/kg/day PB (PHL), HFD + 200 mg/kg/day PB (PHM), and HFD + 400 mg/kg/day PB (PHH). These diets were orally administered daily over a 16-week intervention period, with the N and H groups receiving distilled water as a vehicle control (Figure 1). The food intake and body weight were monitored weekly. The PB doses in this study were based on potentially clinically applicable dosages (0.5, 1.0, and 2 g/60 kg/day) [36]. PB doses (400, 200, and 100 mg/kg) were selected based on prior in vivo efficacy evaluations of other protein hydrolysates in obese animals fed a HFD [37,38,39]. Subsequently, mice were euthanized using 50% isoflurane (Hana Pharm, Seoul, Republic of Korea) diluted in propylene glycol (Daejung Chemicals, Siheung, Republic of Korea), and blood and tissue samples were collected. Blood samples were obtained through cardiac puncture. They were then centrifuged at 3000× *g* for 15 min at 4 °C to isolate the serum, which was subsequently stored at −70 °C until further analysis. Tissues, including the gastrocnemius (GA), quadriceps (QA), tibialis anterior (TA), epididymal white adipose tissue (EAT), subcutaneous white adipose tissue (SAT), and mesenteric white adipose tissue (MAT), were dissected, weighed, and rapidly frozen using liquid nitrogen and kept at −70 °C. GA muscles were fixed with 10% formalin (Sigma-Aldrich, St. Louis, MO, USA) and maintained at room temperature for histological examination. All the animal experiments were conducted in accordance with the guidelines of the Institutional Animal Care and Use Committee of Dankook University (IACUC DKU-22-086).

### 2.3. Serum Biochemical Analysis

Serum insulin levels were determined by enzyme-linked immunosorbent assay (ELISA) using a mouse insulin ELISA kit (Crystal Chem, Zaandam, The Netherlands), and fasting blood glucose levels were quantified using an enzymatic kit (Asan Pharmaceutical Co., Seoul, Republic of Korea). Monocyte chemoattractant protein 1 (MCP-1) and interleukin (IL) 6 levels were quantified by Luminex multiplex assay using the MILLIPLEX MAP Mouse Adipokine Magnetic Bead Panel-Endocrine Multiplex Assay Kit (Millipore, Billerica, MA, USA).

### 2.4. Grip Strength Measurement

Forelimb grip strength was measured after 16 weeks of the dietary intervention using a grip strength testing machine (Bioseb, Chaville, France) [40]. The mice were gently positioned on the grid and a consistent force was applied backward. The maximum grip strength was recorded via five consecutive measurements and normalized to the body weight.

### 2.5. Oral Glucose Tolerance Test (OGTT)

To assess insulin sensitivity, an OGTT was performed at week 15 of treatment (one week prior to euthanasia) by the oral administration of glucose (1 g/kg body weight; Sigma-Aldrich) after 6 h of fasting. Blood glucose concentrations were measured at 0, 15, 30, 60, and 120 min after treatment using an Accu-Chek glucometer (Accu-Chek, Roche, IN, USA). The area under the curve (AUC) of the OGTT results was calculated using the statistical software GraphPad Prism 9.5 (GraphPad, La Jolla, CA, USA). Homeostasis model assessment for insulin resistance (HOMA-IR) was performed using the following formula: HOMA-IR = fasting insulin (ng/mL) × fasting glucose (mg/dL)/405.

### 2.6. Hematoxylin and Eosin Staining

GA muscles were immediately fixed in 10% formalin (Sigma-Aldrich) and embedded in paraffin. Subsequently, the embedded paraffin tissue blocks were cut into 4 μm sections and stained with hematoxylin and eosin [41]. Histological differences were observed in images captured using an optical microscope (BX53; Olympus, Tokyo, Japan) at 40× magnification. The cross-sectional areas of the muscle fibers were determined using ImageJ software (version 1.53, National Institutes of Health, Bethesda, MD, USA) in three different regions per section.

### 2.7. Ectopic Lipid Content

The Folch method was employed to extract total lipids from QA muscle and liver tissues [42,43]. The tissues were homogenized in a 2 to 1 (*v*/*v*) chloroform–methanol mixture and incubated at 4 °C for 1 h. The homogenate was separated by centrifugation at 2400× *g* for 10 min, and a 0.2 volume of 0.88% sodium chloride was added to separate the phases. After the centrifugation, the lower phase was washed and dried at room temperature.

### 2.8. Total RNA Isolation and Quantitative Reverse-Transcription Polymerase Chain Reaction (qRT-PCR)

Total RNA was extracted from the GA muscles using NucleoZOL (Macherey-Nagel, Bethlehem, PA, USA) [44] and reverse-transcribed into cDNA using RocketScript Reverse Transcriptase, RNase H Minus (Bioneer, Daejeon, Republic of Korea) according to the manufacturer’s instructions. Subsequently, the cDNA was amplified and quantified using the CFX Connect Real-Time PCR Detection System (Bio-Rad, Hercules, CA, USA) and iQ SYBR Green Supermix (Bio-Rad). Gene expression levels were expressed as fold changes relative to the H group, and the housekeeping gene (*ribosomal protein lateral stalk subunit P0*) was used for normalization. Relative gene expressions were calculated using the 2^−ΔΔCT^ method. The primer sequences are listed in Table S1.

### 2.9. Western Blotting Analysis

Western blotting was performed as previously described [45]. GA muscles were homogenized in ice-cold radioimmunoprecipitation assay buffer (Elpis Biotech, Daejeon, Republic of Korea) containing a protease inhibitor cocktail (Roche Applied Science, Penzberg, Germany) and a phosphatase inhibitor (Roche). The homogenate was centrifuged for 20 min at 12,000 rpm at 4 °C, and the supernatant was used for protein analysis. Total protein concentration was quantified using the bicinchoninic acid method (Thermo Scientific, Waltham, MA, USA). Subsequently, an identical concentration of protein (20–30 μg/lane) was separated using 10% sodium dodecyl sulfate–polyacrylamide gel electrophoresis and transferred onto a polyvinylidene fluoride membrane (Bio-Rad) at 100 V for 1 h. The membranes were then blocked with 5% skimmed milk (Difco, Detroit, MI, USA) for 1 h at room temperature, followed by incubation in primary antibodies at 4 °C. The membranes were washed with 0.1% Tris-buffered saline containing Tween 20 (Bio-Rad) and then incubated with the corresponding secondary antibodies for 1 h at room temperature. All antibodies used in this study are listed in Table S2. Finally, the immunoreactions were visualized using enhanced chemiluminescence (DoGen Bio, Seoul, Republic of Korea) and imaged using iBright FL750 system (Invitrogen, Carlsbad, CA, USA). Densitometry was performed using the iBright Analysis Software (version 5.2.1, Invitrogen).

### 2.10. Statistical Analyses

Data are expressed as the mean ± standard error of the mean. Statistical analyses were performed using the rank-transformed data. Group comparisons were examined by one-way analysis of variance, followed by Tukey’s post hoc test, with statistical significance set at *p* < 0.05. Outliers with Q values of 10% were excluded using the robust regression and outlier removal method. All analyses were conducted with the statistical software GraphPad Prism 9.5 (GraphPad) [46].

## 3. Results

### 3.1. Effects of PB on Body Weight, Energy Intake, Muscle Weight, and Fat Mass in HFD-Fed Mice

**Figure 2:**
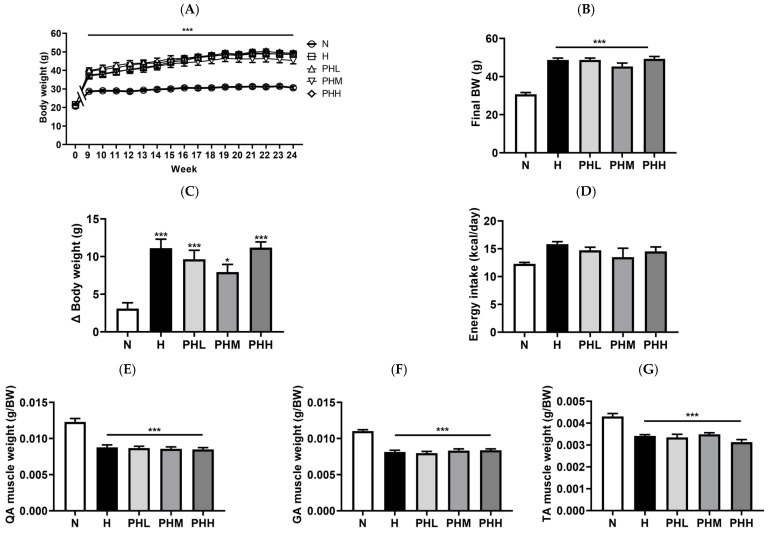

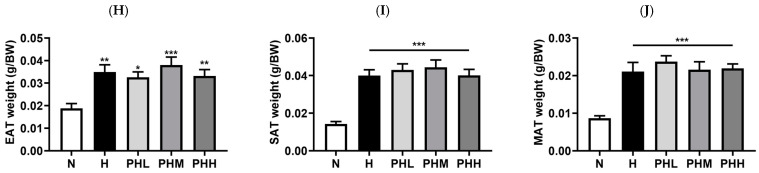
Effect of *Protaetia brevitarsis* (PB) hydrolysate on obesity in high-fat diet (HFD)-fed mice. (**A**) Average body weight curve (*n* = 9–11). (**B**) The body weight measured at the end of the study (*n* = 9–11). (**C**) Body weight gain (*n* = 9–11). (**D**) Final energy intake measured at the end of the study (*n* = 9–11). Ratios of (**E**) quadricep (QA) (*n* = 9–11), (**F**) gastrocnemius (GA) (*n* = 9–11), and (**G**) tibialis anterior (TA) (*n* = 9–11) muscle weights to body weight (g/BW) are shown. Ratios of (**H**) epididymal white adipose tissue (EAT) (*n* = 9–11), (**I**) subcutaneous white adipose tissue (SAT) (*n* = 9–11), and (**J**) mesenteric white adipose tissue (MAT) (*n* = 9–11) weights to body weight (g/BW) are also shown. Data on body weight from 1 to 8 weeks are not indicated. Significant differences in average body weight were observed between all H groups and the N group. No significant differences were noted among the H groups (H, PHL, PHM, and PHH). Data are presented as the mean ± SEM. * *p* < 0.05, ** *p* < 0.01, and *** *p* < 0.001 represent the significant differences compared to the N group. BW, body weight; N, normal chow diet; H, HFD; PHL, HFD + 100 mg/kg PB; PHM, HFD + 200 mg/kg PB; PHH, HFD + 400 mg/kg PB.

The nutraceutical potential of PB hydrolysate for HFD-mediated metabolic disorders induced by HFD was evaluated by administering PB hydrolysate at varying doses (100, 200, and 400 mg/kg) to obese mice on HFD over a 16-week period (Figure 2). After eight weeks of the HFD, a 25% increase in body weight confirmed diet-induced weight gain (Figure 2A). Although PB supplementation did not significantly lower the body weight gain induced by the HFD at the conclusion of the study (Figure 2B), the group receiving 200 mg/kg PB (PHM group) exhibited a 28% decrease in body weight gain relative to the H group (Figure 2C). Notably, the energy intake was consistent in all groups (Figure 2D). Muscle weight was markedly lower in the H group than that in the N group, and PB supplementation did not prevent this reduction (Figure 2E–G). The SAT, EAT, and MAT weights were substantially greater in group H than in group N (Figure 2H–J). However, 16 weeks of PB supplementation did not alter subcutaneous or visceral fat mass in an HFD-induced metabolic syndrome model.

### 3.2. Effect of PB Hydrolysate on the Insulin Sensitivity of HFD-Fed Mice

**Figure 3:**
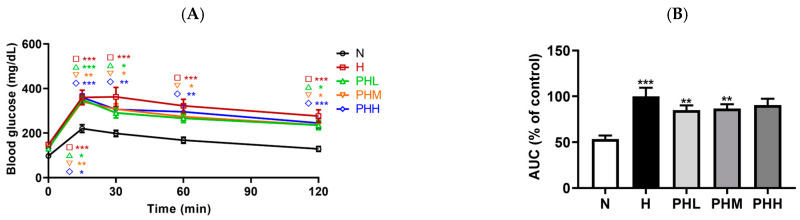

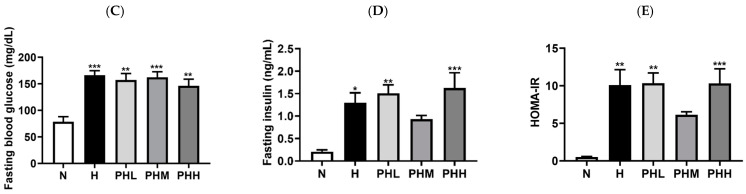
Effect of PB hydrolysate on the insulin sensitivity of HFD-fed mice. (**A**) Time-based blood glucose levels measured from the oral glucose tolerance test (OGTT) curve (*n* = 9–11). OGTTs were conducted at week 15 of PB intervention. (**B**) Area under the curve (AUC) of the OGTT curve (*n* = 9–11). (**C**) Fasting blood glucose levels at the conclusion of the study (*n* = 9–11). (**D**) Fasting insulin levels at the end of the conclusion of the study (*n* = 8–11). (**E**) Homeostasis model assessment for insulin resistance (HOMA-IR) values at the conclusion of the study (*n* = 7–11). Data are presented as the mean ± SEM. * *p* < 0.05, ** *p* < 0.01, and *** *p* < 0.001 represent the significant differences compared to the N group. N, normal chow diet; H, HFD; PHL, HFD + 100 mg/kg PB; PHM, HFD + 200 mg/kg PB; PHH, HFD + 400 mg/kg PB; HOMA-IR, homeostasis model assessment for insulin resistance.

The OGTT was performed during the last week of the experiment for a total of 120 min (Figure 3A). The OGTT results showed 20% and 22% reductions in blood glucose levels at 30 and 60 min, respectively, in the low-dose PB (PHL) group relative to the H group; however, these reductions were not statistically significant (Figure 3A). Additionally, the blood glucose levels at 60 min showed a decreasing tendency in the mid-dose PB (PHM) group by 20% in the PHM group (Figure 3A). After 24 weeks of HFD feeding, AUC for the OGTT was elevated in the H group compared to the N group. However, the AUC in the PB hydrolysate-supplemented groups was not significantly different from that in the H group (Figure 3B). PB supplementation did not affect fasting blood glucose levels (Figure 3C). Nevertheless, the PHM group showed a 28% decrease in fasting insulin levels relative to the H group, reflecting a 34% improvement in insulin sensitivity based on HOMA-IR (Figure 3D,E). These findings suggested that the mid-dose PB supplementation enhanced insulin sensitivity in HFD-fed mice.

### 3.3. Effect of PB Hydrolysate on HFD-Induced Muscular Dysfunction

**Figure 4:**
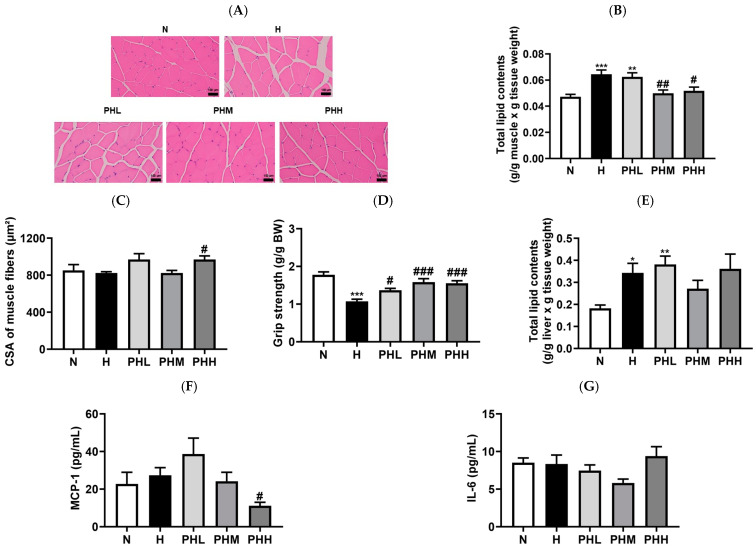
Effect of PB hydrolysate on HFD-induced muscular dysfunction. (**A**) Representative images of hematoxylin and eosin (H&E) staining (*n* = 9–11) and (**B**) Total lipid contents of the QA muscle (*n* = 9–11). (**C**) The cross-sectional area (CSA) of muscle fibers in the GA muscle (*n* = 9–11). The magnification was 40×. (**D**) Forelimb grip strength at 16 weeks with or without PB supplementation (*n* = 9–11). (**E**) Total lipid contents of the liver (*n* = 8–11). Levels of (**F**) monocyte chemoattractant protein (MCP) 1 (*n* = 8–11) and (**G**) interleukin (IL) 6 (*n* = 7–11) secreted into the blood with or without PB supplementation for 16 weeks. Data are presented as the mean ± SEM. * *p* < 0.05, ** *p* < 0.01, and *** *p* < 0.001 represent the significant differences compared to the N group. # *p* < 0.05, ## *p* < 0.01, and ### *p* < 0.001 represents the significant differences compared to the H group. BW, body weight; N, normal chow diet; H, HFD; PHL, HFD + 100 mg/kg PB; PHM, HFD + 200 mg/kg PB; PHH, HFD + 400 mg/kg PB; GA, gastrocnemius; CSA, cross-sectional area; MCP-1, monocyte chemoattractant protein-1; IL, interleukin.

The effects of PB hydrolysates on HFD-induced muscle wasting were explored by assessing ectopic lipid levels in skeletal muscle (Figure 4A,B). Prolonged HFD intervention generally has deleterious effects on skeletal muscle mass, strength, and function by increasing fatty acid infiltration, leading to skeletal muscle insulin resistance [47]. In this study, lipid accumulation in skeletal muscle significantly increased after HFD intake (Figure 4A,B). However, the PB hydrolysate significantly attenuated IMAT deposition in the mid- and high-dose PB groups. Corresponding to the reduction in IMAT content, high-dose PB supplementation significantly increased muscle fiber size compared to the H group (Figure 4C). Additionally, PB supplementation significantly restored muscle strength, which was impaired by prolonged HFD consumption (Figure 4D). Obesity-related adipose tissue dysfunction often results in hepatic lipid accumulation [48]. Therefore, we examined the effect of PB on hepatic lipid accumulation, a key factor regulating overall glucose and lipid metabolism [49]. The hepatic lipid levels were markedly higher in the H group than in the N group (Figure 4E). However, the PHM group exhibited a 30% reduction in hepatic lipid accumulation relative to that in the H group, although this difference was not statistically significant (Figure 4E). Reduced ectopic fat deposition in the muscles and liver following PB supplementation has been linked to the amelioration of systemic low-grade chronic inflammation. High-dose PB supplementation significantly reduced serum MCP-1 levels (Figure 4F). Moreover, systemic IL-6 levels were reduced by 30% in the PHM group (Figure 4G).

### 3.4. Effect of PB Hydrolysate on Skeletal Muscle Lipid Metabolism in HFD-Fed Mice

**Figure 5:**
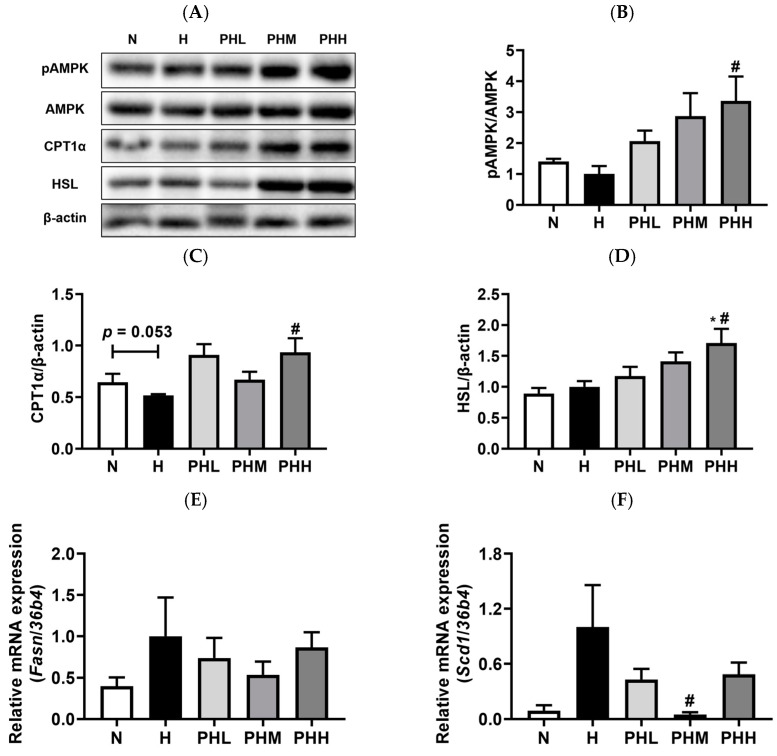
Effects of PB hydrolysate on gene and protein expressions in the GA muscle. (**A**) Representative Western blot band images. Quantification of bands: (**B**) phospho-AMP-activated protein kinase (AMPK) (*n* = 4), (**C**) carnitine palmitoyltransferase (CPT) 1α (*n* = 9–11), and (**D**) hormone-sensitive lipase (HSL) (*n* = 9–11). Quantitative reverse-transcription-polymerase chain reaction (qRT-PCR) validation of (**E**) fatty acid synthase (*Fasn*) (*n* = 8–11) and (**F**) stearoyl-CoA desaturase 1 (*Scd1*) (*n* = 7–11) levels in GA muscles. Data are presented as the mean ± SEM. * *p* < 0.05 represents the significant differences compared to the N group. # *p* < 0.05 represents the significant differences compared to the H group. N, normal chow diet; H, HFD; PHL, HFD + 100 mg/kg PB; PHM, HFD + 200 mg/kg PB; PHH, HFD + 400 mg/kg PB; pAMPK, phosphorylated AMP-activated protein kinase, AMPK, AMP-activated protein kinase; CPT1α, carnitine palmitoyltransferase 1 alpha; HSL, hormone-sensitive lipase; FASN, fatty acid synthase; SCD1, stearoyl-coenzyme A desaturase 1.

Integrated transcript and protein analyses of skeletal muscles were conducted to elucidate the mechanisms of action of the PB hydrolysate on skeletal muscles (Figure 5). Phosphorylated AMPK levels were found to be significantly and dose-dependently increased in the PHH group, and levels of CPT1α, a protein associated with fatty acid oxidation, were upregulated with high-dose PB supplementation (Figure 5A–C). The levels of hormone-sensitive lipase (HSL), a key enzyme for lipolysis, were elevated in the PHH group, indicating that PB induced lipolysis in IMAT (Figure 5D). At the transcriptional level, fatty acid synthase (*Fasn*) gene levels, which were elevated in the HFD group, decreased by 46% in the PHM group (Figure 5E). Similarly, stearoyl-CoA desaturase 1 (*Scd1*) levels, which were elevated in the HFD group, were significantly reduced following PB supplementation (Figure 5F). These findings suggested that PB modulates lipid metabolism, reduces IMAT accumulation, and enhances muscle function in individuals with HFD-induced metabolic syndrome.

## 4. Discussion

Metabolic syndrome encompasses a combination of conditions, including hypertension, visceral obesity, dyslipidemia, and elevated fasting glucose, which collectively increase the risk of diabetes and cardiovascular diseases [1,50]. Obesity is often the initial driver of metabolic syndrome and exacerbates chronic low-grade inflammation [4]. A high-fat and high-calorie diet plays a significant role in the onset of metabolic disorders [51]. As demonstrated in this study, the HFD-fed mice experienced notable gains in body weight, fat mass, and insulin resistance, which were often accompanied by disruptions in muscular function. Notably, although PB supplementation did not significantly alter body weight, fat mass, or HOMA-IR levels compared to the H group, PB supplementation effectively alleviated muscular dysfunction and systemic inflammation through AMPK-mediated regulation of lipid metabolism in skeletal muscles. Our findings demonstrate that PB supplementation provides significant metabolic and muscular benefits without altering body weight or insulin resistance, suggesting a targeted mechanism of AMPK activation that may offer preventive advantages in managing metabolic syndrome.

Previous studies have established that PB has a high protein content, ranging from 51.16% to 67.07%, and includes essential amino acids such as leucine, isoleucine, histidine, methionine, lysine, phenylalanine, tryptophan, and valine, as well as non-essential amino acids such as glutamic acid and proline [26,27]. The enzymatic hydrolysis of PB generates bioactive peptides with diverse functionalities, including antimicrobial, antihypertensive, antioxidant, hepatoprotective, and hypolipidemic effects [28,33,52]. PB also produces secondary metabolites, such as dopamine, quinoxaline, and diketopiperazine, which play a role in its pharmacological activity [32,53,54]. PB polyphenols have potent antioxidant and anti-inflammatory properties, which are essential for improving metabolic health [23,24]. These bioactive components likely underlie the observed therapeutic benefits of the PB hydrolysate.

In this study, mid-dose PB supplementation resulted in notable improvements in insulin sensitivity and a decrease in ectopic lipid accumulation within the skeletal muscle, which is a key factor in ameliorating insulin resistance. Enhanced muscle strength is correlated with reduced lipid infiltration and improved insulin signaling. Our results align with those of previous studies, demonstrating the pivotal importance of skeletal muscle health in controlling systemic glucose metabolism [10]. Dysregulated lipid metabolism and inflammatory responses, often driven by ectopic fat deposition, were effectively mitigated by PB supplementation. Upregulation of AMPK, an essential regulator of energy homeostasis, highlights the mechanistic basis of these benefits. Elevated expression of CPT1α and HSL further supports how PB enhances fatty acid oxidation and lipid metabolism.

Dose-dependent effects of PB supplementation were evident in this study, highlighting the nuanced impact of varying doses. Mid-dose PB supplementation significantly improved insulin sensitivity, muscle strength, and lipid metabolism. These changes were accompanied by reductions in fasting insulin levels and ectopic lipid accumulation. High-dose PB supplementation, while associated with similar improvements, provided additional benefits, such as increased muscle fiber size and enhanced AMPK activation. However, high-dose supplementation also led to a mild increase in systemic IL-6 levels, which may indicate stimulation of myokine secretion. Myokines, including IL-6, promote fatty acid oxidation and enhance energy metabolism via AMPK-dependent signaling pathways [55]. These results indicate that PB supplementation exhibits dose dependence, with mid- and high-dose regimens offering complementary therapeutic benefits. Optimizing the dosage of PB is crucial for maximizing its efficacy while minimizing its potential side effects.

The role of PB hydrolysates in lipid metabolism is more pronounced in skeletal muscles than in hepatic tissues. Although the hepatic lipid content was reduced in the mid-dose group, these changes were not statistically significant. Skeletal muscle demonstrated a significant reduction in ectopic lipid accumulation and enhanced lipid metabolism, suggesting that PB hydrolysates preferentially target muscle tissues for lipid-lowering effects. Muscle-specific ectopic fat reduction may be due to the high metabolic activity of skeletal muscles and their critical role in energy utilization. Downregulation of lipogenic genes, including *Fasn* and *Scd1*, observed in the skeletal muscle further supports our hypothesis. These findings suggest that PB hydrolysates have tissue-specific effects, with the skeletal muscle serving as the primary site of metabolic improvement while maintaining hepatic integrity.

This study had several limitations that should be addressed to better understand and validate our findings. First, this study was conducted using a murine model, and further investigation is necessary to determine the translational applicability of these results to human populations. In addition, the specific bioactive compounds responsible for the observed effects should be identified in future studies. Future studies should focus on isolating and characterizing these compounds to better understand their underlying mechanisms of action. The long-term safety and efficacy of PB supplementation should be evaluated in a broader and more diverse population to confirm its potential as a nutraceutical agent.

## 5. Conclusions

In summary, this study demonstrates the therapeutic potential of PB in mitigating metabolic syndrome by decreasing ectopic fat deposition and enhancing skeletal muscle function. Our findings revealed the roles of PB in regulating lipid metabolism and inflammatory responses via AMPK activation, highlighting the use of PB hydrolysate as a promising strategy to manage metabolic disorders. Future studies should aim to optimize the dosage, elucidate the bioactive components, and validate the effects of PB in clinical settings. Overall, this study provides a foundation for the development of novel PB-based interventions as sustainable and effective solutions for managing metabolic health.

## Data Availability

The original contributions presented in this study are included in the article/Supplementary Material. Further inquiries can be directed to the corresponding authors.

## Supplementary Materials

The following supporting information can be downloaded from https://www.mdpi.com/article/10.3390/nu17020213/s1. Table S1, Primers for qRT-PCR; Table S2, List of antibodies used for Western blot analysis.
