# Supplementary material for: Protaetia brevitarsis Hydrolysate Mitigates Muscle Dysfunction and Ectopic Fat Deposition Triggered by a High-Fat Diet in Mice
Source: Nutrients. 2025 Jan 8;17(2):213. doi: 10.3390/nu17020213 (PMC11767481; doi:10.3390/nu17020213)
Supplement: Supplementary file 1 [file nutrients-17-00213-s001.zip › nutrients-3403130-supplementary.pdf]

Table S1. Primers for qRT-PCR

Primer	Sequences (5' – 3')
Fasn-F	CACAGTGCTCAAAGGACATGCC
Fasn-R	CACCAGGTGTAGTGCCTTCCTC
Scd1-F	GCAAGCTCTACACCTGCCTCTT
Scd1-R	CGTGCCTTGTAAGTTCTGTGGC
36b4-F	GCTTCGTGTTCACCAAGGAGGA
36b4-R	GTCCTAGACCAGTGTTCTGAGC

Abbreviations: *36b4*, Ribosomal Protein Lateral Stalk Subunit P0; *Fasn*, Fatty acid synthase; F, Forward; R, Reverse; *Scd-1*, Stearoyl-CoA desaturase-1.

Table S2. List of antibodies for western blot analysis

Primary Antibodies	Company	Cat. No.	Dilution
Phospho-AMPKα (Thr172)	Cell Signaling	2535	1:1000
AMPKα	Cell Signaling	2532	1:1000
CPTI	Santa Cruz	Sc-393070	1:200
HSL	Cell Signaling	18381	1:1000
β-actin	Santa Cruz	Sc-47778	1:500
**Secondary Antibodies**	**Company**	**Cat. No.**	**Dilution**
Anti-mouse IgG, HRP-linked Antibody	Cell Signaling	7076	1:2000
Anti-rabbit IgG, HRP-linked Antibody	Cell Signaling	7074	1:2000

Abbreviations: AMPK, 5' adenosine monophosphate-activated protein kinase; CPTI, Carnitine palmitoyltransferase I; HRP, Horseradish peroxidase; HSL, Hormone-sensitive lipase.
